# RDoC Framework Through the Lens of Predictive Processing: Focusing on Cognitive Systems Domain

**DOI:** 10.5334/cpsy.119

**Published:** 2024-10-30

**Authors:** Anahita Khorrami Banaraki, Armin Toghi, Azar Mohammadzadeh

**Affiliations:** 1 Institute for Cognitive Science Studies, Tehran, Iran; 2 Institute for Cognitive and Brain Sciences, Shahid Beheshti University, Tehran, Iran; 3 Research Center for Cognitive and Behavioral Studies, Tehran University of Medical Science, Tehran, Iran

**Keywords:** Research domain criteria, Predictive processing, Predictive coding, Active inference, computational psychiatry, cognition, RDOC

## Abstract

In response to shortcomings of the current classification system in translating discoveries from basic science to clinical applications, NIMH offers a new framework for studying mental health disorders called Research Domain Criteria (RDoC). This framework holds a multidimensional outlook on psychopathologies focusing on functional domains of behavior and their implementing neural circuits. In parallel, the Predictive Processing (PP) framework stands as a leading theory of human brain function, offering a unified explanation for various types of information processing in the brain. While both frameworks share an interest in studying psychopathologies based on pathophysiology, their integration still needs to be explored. Here, we argued in favor of the explanatory power of PP to be a groundwork for the RDoC matrix in validating its constructs and creating testable hypotheses about mechanistic interactions between molecular biomarkers and clinical traits. Together, predictive processing may serve as a foundation for achieving the goals of the RDoC framework.

## 1. Introduction

Despite recent advances in neuroscience, molecular biology, and cognitive science, much is still unknown about the brain mechanisms behind psychiatric disorders (Scangos et al., 2023; Willsey et al., 2018). The current categorization system, including the Diagnostic and Statistical Manual of Mental Disorders DSM-5-TR (American Psychiatric Association, 2022), and the Mental and Behavioral Disorders section of the International Classification of Diseases ICD (World Health Organization, 2019), does not map well into the emerging findings from genetics, system neuroscience, and behavioral science (Cohen & Öngür, 2023); consequently, there is no such clear path in translating research from primary studies, like in animal models and humans, to a clear understanding of psychopathologies or systematic treatments which target the related mechanism (Cuthbert & Insel, 2013).

NIMH’s research domain criteria (RDoC) project, initiated in 2009, intended to use a multidimensional approach, focusing on translational research on functional domains of behavior or psychological processes across the range of functioning from normal to abnormal (Cuthbert, 2020). The organization of the RDoC matrix comprised of six functional domains (Negative Valence Systems, Positive Valence Systems, Cognitive Systems, Social Processes, Arousal and Regulatory Systems, and Sensorimotor Systems) integrated into different units of analysis embracing genes, molecules, circuits, physiology, behavior, and self-report with consideration of environmental and developmental factors (Cuthbert, 2020; S. E. Morris et al., 2022). RDoC aims to enhance the translation of circuit-level knowledge about psychiatric disorders from basic science to clinical practice, seeking to identify specific neural targets and adopt a more mechanistic and targeted approach to treatment development (Scangos et al., 2023). For a comprehensive discussion about the RDoC framework, see; (Cuthbert, 2022; S. E. Morris et al., 2022).

For this ambitious goal, RDoC needs a mechanistic understanding of the main biological components involved in psychopathologies, their relations to behavioral changes in mental illnesses, and the reason behind these changes (Simmons et al., 2020). The emerging field of computational psychiatry employs mathematical or computational models of brain function to understand and describe the underlying mechanisms of psychopathologies (K. J. Friston et al., 2014). These models provide a formal framework for analyzing and characterizing psychopathological processes using computational and mathematical terms (K. J. Friston et al., 2014).

These computational models are divided into two broad groups: well-defined theory-driven approaches and exploratory data-driven models (Huys et al., 2016; Simmons et al., 2020). Theory-driven approaches utilize models that incorporate prior knowledge or explicit hypotheses about the mechanisms, potentially at various levels of analysis and abstraction (Huys et al., 2016). Conversely, Machine-learning methods are employed in data-driven approaches to enhance disease classification, treatment outcome prediction, and treatment selection using high-dimensional datasets (Huys et al., 2016). These two approaches are highly complementary and promising.

RDoC funding projects prioritize multisystem integration, encouraging scientists to use these computational models to evaluate and validate RDoC constructs through quantitative analysis of the relationships between various measurement systems (Cuthbert, 2022). In other words, how these constructs are segregated, overlapped, or interrelated in terms of their underlying neural circuits would be assessed by theory/data-driven computational models.

Theory-driven approaches utilize formal models to give us a mechanistic understanding of brain/behavior relationships that serve as an excellent tool for validating RDoC construct and integrating units of analysis in a meaningful way (Ferrante et al., 2019). Moreover, the collection and interpretation of data-driven approaches depend on a theoretical background; reciprocally, theory-driven models need data to test their plausibility (Ferrante et al., 2019).

In the 2017 NIMH workshop for opportunities and challenges of computational psychiatry, participants highlighted the importance of working toward a “common language” about the underlying computational theories of mental constructs (Ferrante et al., 2019). Here we talk in favor of the predictive processing (PP) framework. PP framework is an umbrella term for different theory-driven computational models that explain various brain functions in terms of prediction and prediction error minimization (e.g., predictive coding and active inference). Although evidence supporting these models is considered as evidence for a broader idea of PP, they differ in algorithmic and implementation level (Hodson et al., 2024).

This framework encompasses theoretical models that can stretch from cellular biology to phenomenology that could bring experts in a variety of scales (e.g., molecular biologists, clinical neuroscientists) to converge their findings in one unifying concept that finally leads to explaining psychopathology in terms of pathophysiology (K. Friston, 2023).

Based on this framework, any symptom of psychopathology, at some level, arises from false inference (K. Friston, 2023). False inference is attributed to the imbalance in message passing between prediction and prediction error units at different levels of the hierarchy. Crucially, this imbalance is attributed to aberrant precision weighting of hierarchical prediction errors. This provides a link between belief updating and pathophysiology; in the sense that precision weighting is thought to be mediated by neuromodulatory effects. In turn, this speaks to a pernicious (neuromodulatory) synaptopathy, consistent with a view of psychiatric disorders as functional dysconnection syndromes (K. J. Friston et al., 2014).

Empirically, precision weighting imbalance is suggested to explain many psychiatric conditions like autism (Lawson et al., 2017; Van de Cruys et al., 2014), ADHD (Richards et al., 2020), psychosis (Adams et al., 2013; Powers et al., 2017; Sterzer et al., 2018), PTSD (Homan et al., 2019), anxiety (Hein et al., 2023; Paulus et al., 2019), personality disorders (Moutoussis et al., 2014), and depression (Badcock et al., 2017).

Indeed, PP models can establish a clear connection between neural systems and behavior (Ferrante et al., 2019; K. Friston, 2023; Huys et al., 2016; Shine et al., 2021). This allows scientists to develop solid theoretical conceptualizations that establish bidirectional links across different units of analysis, ranging from molecules to circuits and from circuits to behavior (Ferrante et al., 2019).

Here, we suggested that an explanatory power of the PP framework could serve as a groundwork for the RDoC matrix. Equally, PP benefits more if it characterizes psychopathologies multi-dimensionally (e.g., autism-schizophrenia continuum (Tarasi et al., 2022)).

In the first section, we foreground empirical evidence of the PP framework that presents a mesoscale understanding of the normative neurobehavioral functions listed in constructs within the cognitive system domain of RDoC. This section only focuses on studies involving healthy human participants, where we bring theoretical explanations of the PP framework and subsequent empirical evidence supporting those explanations. We restrict our review article on the cognitive construct of RDoC due to the extensive basic science research conducted on these psychological processes. Moreover, we only consider studies with human participants, as RDoC emphasized for validating its construct (NIMH 2024).

After this section, we turned to PP’s explanatory potential in understanding psychopathologies, especially, to bring a mechanistic understanding of connections between biomarkers and clinical traits in the psychosis continuum by targeting particular RDoC construct (Perception). Lastly, we provide a framework to illustrate how these two lines of research can be integrated, guiding future directions in this field.

## 2. Predictive Processing Framework in the Cognitive System Domain of RDoC

RDoC offers a translational perspective in studying mental health disorders, starting with what we know about normative neurobehavioral functions (for example, what we know about attention?), and mental health disorders were studied as disruptions in these functions leading to dysfunction of varying degrees (S. E. Morris et al., 2022). Meanwhile, there is still a lack of understanding of the brain function underlies these constructs, mainly due to the complexity of studying brain circuits underlies a specific type of information processing referred to as mesoscale.

Predictive processing casts brain function as belief updating in the face of new information to maximize the evidence for internal or generative world models. This affords a powerful framework for linking microscale (i.e., cellular and molecular function) and macroscale (i.e., behavioral and self-reports) (Smith et al., 2021).

This section will briefly examine recent advancements in PP hypotheses and empirical evidence from basic science related to the constructs within the cognitive system domain, including perception, attention, working memory, language, and declarative memory.

### 2.1. Perception

Perception involves a series of complex processes through which we receive information from our senses, organize and interpret it, and give it meaning (Pomerantz, 2006). The perception construct of RDoC is further divided into Visual, Auditory, and Olfactory/Somatosensory/Multimodal subconstructs.

In terms of PP, perception arises from a bidirectional message passing between hierarchical cortical levels; ascending prediction error is thought to be represented explicitly by superficial pyramidal cells, and descending prediction is thought to originate in deep pyramidal cells that cancel prediction error via targeting inhibitory interneurons that are connected with superficial pyramidal cells (Bastos et al., 2012; Shipp, 2016). Moreover, prediction errors are modulated by precision (predicting precision) via modulatory backward connections dealing with context dependencies (K. Friston, 2018).

Empirical evidence for supporting PP in early visual processing primarily arises from studies observing early visually evoked responses in the absence of bottom-up input, across both deep and superficial layers of primary visual cortex (V1) (Aitken et al., 2020; Kok et al., 2016; Muckli et al., 2015). Ultra-high field fMRI studies showed, prior expectations selectively trigger stimulus-specific activity in the deep layers of the V1 (Aitken et al., 2020), while unexpected events invoke responses in superficial layers of V1 (Thomas et al., 2024). These findings support the PP laminar specification of prediction and prediction error in deep and superficial layers of early visual processing, respectively.

In higher-order visual processing, PP is mainly supported by expectation suppression paradigms (Hodson et al., 2024), indicating that expected or predicted stimuli evoke smaller responses. In the Egner et al. (2010) experiment, participants responded to expected and unexpected face and house stimuli. The study found that when faces and objects are highly expected, the BOLD activity in the fusiform face area (FFA) was indistinguishable; however, with lower expectation levels, the FFA’s response to faces was greater than objects. Given the central role of the FFA in face processing, the strong response to unexpected faces (rather than objects), can be well explained by the presence of prediction error units in category-specific visual areas such as the FFA (Egner et al., 2010).

In the auditory sub-construct, support for PP mostly comes from auditory mismatch negativity (MMN) paradigms (see (Heilbron & Chait, 2018)). These paradigms typically consist of repeated tone sequences disrupted by an atypical deviant tone. MMN is calculated by subtracting the brain’s response to the standard tone from its response to the deviant (Heilbron & Chait, 2018). This physiological component is considered an RDoC element in auditory perception and interpreted as a prediction error (i.e., the discrepancy between sensory inputs and predictions) within the PP framework.

Dynamic causal modeling (DCM) is a prevalent approach extensively used to test PP in relation to auditory MMN (Garrido et al., 2009; Heilbron & Chait, 2018). DCM studies of evoked potentials in different oddball paradigms revealed that frequency, intensity, and duration MMNs are best explained with bidirectional connectivity changes between primary auditory cortex (A1), inferior frontal gyrus (IFG), and superior temporal gyrus (STG) (Garrido et al., 2009). Garrido et al. (2008) tries to compare three competing theories of MMN generation, including adaptation, memory adjustment, and predictive coding using DCM of evoked potentials. The results show that the predictive coding model, incorporating elements of both adaptation and model adjustment, best explained the ERP differences (Garrido et al., 2008). Complementary, evidence for the predictive nature of top-down auditory signals is also provided by omission paradigms (Heilbron & Chait, 2018; Hodson et al., 2024), which shows that omitting an expected sound can still evoke brain responses time-locked to the omitted stimulus (Heilbron & Chait, 2018).

Moreover, mismatch responses at shorter latencies than the traditional MMN indicate potential involvement of sub-cortical auditory pathways in PP processes (Cacciaglia et al., 2015; Escera, 2023). Cacciaglia et al. (2015) explored this by examining BOLD responses in a passive frequency oddball paradigm, supporting the role of sub-cortical auditory pathways including the inferior colliculus (IC) and medial geniculate body (MGB) in statistical inference and regularity encoding.

Brain rhythms are another approach to understanding the PP underlying visual and auditory perception (Walsh et al., 2020). Much work in human and primate studies supports the cortical communication between alpha/beta feedback connections from deep cortical layers and gamma feedforward connections in superficial cortical layers (Fontolan et al., 2014; Mendoza-Halliday et al., 2024). From the predictive coding perspective, alpha/beta feedback connections carry predictive information, and prediction errors are related to gamma-band oscillations (Bastos et al., 2012). This statement has repeatedly been supported in human studies utilizing Electrocorticography (ECoG) (Dürschmid et al., 2016; Edwards et al., 2005; El Karoui et al., 2015; Sedley et al., 2016), EEG (Chao et al., 2022; Mohanta et al., 2021), and Magnetoencephalography (MEG) (Arnal et al., 2011).

In the «Olfactory/Somatosensory/Multimodal Perception» sub-construct, PP is supported by limited but significant evidence. This includes the observed predictive activity in the piriform cortex (PPC) during olfactory search task (Zelano et al., 2011), as well as predictive feedback mechanisms observed in both superficial and deep layers of the somatosensory cortex during prediction tasks (Yu et al., 2019). Additionally, there are emerging concepts regarding the role of PP in multisensory integration, suggesting its broader applicability across various sensory modalities (Talsma, 2015).

Based on comprehensive review articles that evaluate PP claims in perception in a series of invasive and non-invasive studies, it seems some of the PP claims like hierarchically organized predictions underlying perception are well supported (Heilbron & Chait, 2018; Hodson et al., 2024; Walsh et al., 2020). However, the empirical data supporting the existence of separate prediction and prediction error units still need to be established (Heilbron & Chait, 2018; Walsh et al., 2020).

### 2.2. Attention

Attention function serves several purposes, such as maintaining a state of alertness, picking out relevant information from sensory input, and regulating conflicts (Posner & Rothbart, 2007). The attention networks consist of dispersed computational nodes located in various brain regions that often collaborate with networks responsible for sensory perception, memory, and various other functions (Posner, 2023; Posner & Rothbart, 2023).

Attention in the PP framework is a function for optimizing perception and learning via collecting contextually informative sensations (Lecaignard et al., 2022; Parr & Friston, 2019). This could happen through the precision weighting of sensory channels or actions (Lecaignard et al., 2022). In other words, attending to the features of a stimulus is equivalent to predicting high precision for related prediction error that increases the influence of that error for updating related perceptual hypothesis (Walsh et al., 2020), or in the case of active inference; it corresponds to a behaviorally salient action for reducing uncertainty (Parr & Friston, 2019). However, the physical implementation of precision modulation is one of the less comprehensively understood aspects of PP (Sprevak & Smith, 2023). Theoretically, precision modulation is suggested to occur via the neuromodulatory mechanism of gain control at a synaptic level (Moran et al., 2013), or fast synchronized presynaptic inputs (Feldman & Friston, 2010).

Recent Meta-analyses of functional connectivity studies based on predictive coding show remarkable similarities between brain regions involved in prediction and brain networks associated with top-down control of attention (e.g., dorsal attention network) (Ficco et al., 2021). Meanwhile, a growing body of evidence supports the dissociable yet intertwined roles of attention and prediction in cognitive processes (Auksztulewicz & Friston, 2016; Ficco et al., 2021; Hsu et al., 2014; Kok et al., 2012), which shows voluntary attention improves the precision of perceptual inference by up-weighting prediction error signals (Garrido et al., 2018).

Even without voluntary attention, a stable or predictable environment facilitates efficient learning through an implicit precision-weighted process (Lecaignard et al., 2022; Rowe et al., 2023). Lecaignard et al. (2022) showed this by using simultaneous EEG-MEG recording while participants performed the passive auditory oddball task. By manipulating sound predictability and using trial-by-trial modeling of cortical responses and the DCM of evoked responses, they also found empirical evidence for the link between precision weighting of prediction errors and self-inhibition in superficial pyramidal cells. They argued that linking voluntary attention and the passive predictability process would be a promising way to investigate attentional capture mechanistically.

In sum, the attention function is suggested to emerge from precision modulation via modulating post-synaptic gain at different levels of the hierarchy, and attention networks are hypothesized to have a crucial role in estimating the precision of prediction signals (Katsumi et al., 2023).

### 2.3. Working memory

Working memory deals with the selective maintenance and manipulation of information when we are not exposed to external stimuli (Baddeley, 2011). There are few simulations, and empirical evidence tries to conceptualize the working memory function and its interaction with other cognitive processes (e.g., decision-making and attention) under the assumptions of PP.

The frontal lobe plays a vital role in working memory function (Prabhakaran et al., 2000). Alexander and Brown (2018) propose a simple computational motif for frontal cortex function referred to as the Hierarchical Error Representation (HER) model. In their model, the error signal in mPFC train representation of the error signal in dlPFC. Then, this error is learned and maintained in dlPFC for reducing prediction error in mPFC for subsequent stimulus presentation. The simulation of this model in a variety of findings, including fMRI, ERP, single-unit, and neuropsychological studies, shows that this self-organized hierarchical network could learn, maintain, and flexibly change working memory representation (as a product of learning) for prediction error minimization (Alexander & Brown, 2018).

Simulation studies based on active inference models conceptualized working memory function as an accumulation of evidence within temporal hierarchies (Parr & Friston, 2017; Parr et al., 2020), that involves evaluating future policies or accumulating evidence for different stages of the world to predict future states and guide decision-making (Parr & Friston, 2017). In this conceptualization, updating or maintenance of representations in working memory depends on attentional processes. In that sense, updating working memory involves perceiving sensory data as precise while maintaining a representation in the presence of distractions requires perceiving new sensory data noisy (Parr & Friston, 2017). Although the model has been evaluated through simulated ERP, electrophysiological, and in silico lesion experiments (Parr & Friston, 2017; Parr et al., 2020), we have not discovered any empirical evidence directly supporting this explanation.

### 2.4. Declarative memory

‘Declarative’ or conscious memories refer to memories of facts and events that are consciously available. This function highly depends on the hippocampus in the brain’s temporal lobe (Hainmueller & Bartos, 2020). The hippocampus plays a fundamental role in all processing stages of learning (e.g., memory encoding, consolidation, and retrieval) (Topolnik & Tamboli, 2022) and also has a remarkable capacity for online prediction of upcoming sensory inputs (Barron et al., 2020). From a PP perspective, the hippocampus is crucial in learning environmental statistics and exploits them for generating perceptual predictions (Aitken & Kok, 2022; Katsumi et al., 2023; Pezzulo et al., 2017).

Firstly, memory encoding and retrieval through pattern separation and completion have suggested relying on prediction error, in which, in the encoding phase, prediction error derives learning to update our internal model of the world, while in retrieval mode, we have learned the statistical regularities of the environment; thus, prediction error decrease and predictions may dominate (Aitken & Kok, 2022; Bein et al., 2020; Henson & Gagnepain, 2010). Bein et al. (2020) showed that when human participants face novel stimuli, prediction error drives the hippocampus towards an encoding mode with increasing CA1-entorhinal connectivity and stops a retrieval mode through decreasing CA1-CA3 connectivity. Recently, Aitken and Kok (2022) conducted an fMRI study to illustrate how the hippocampus balances encoding and retrieval in a predictive association task. In this study, they demonstrated that the hippocampus switches from representing prediction error (encoding mode) during learning to represent prediction when the learning processes are completed.

Secondly, declarative memory function occurs via cortico-hippocampal and cortico-cortical interactions. Barron et al. (2020) proposed a PP version of neocortical-hippocampal interaction based on long-range inhibitory pathways. They suggested that the hippocampus projects prediction via long-range GABAergic neurons to explain away activity in lower-level regions (Barron et al., 2020). A recent ultra-high field fMRI study supported this idea by showing a negative predictive representation in CA2/CA3 and deep layers of the parahippocampal cortex while participants performed an omission task (Warrington et al., 2024). Complementary, a DCM study supports the role of vmPFC in driving the hippocampal theta during the processing of prediction violation signals (Garrido et al., 2015). These support the role of hippocampus in explaining away predicted ascending cortical inputs and neocortical-hippocampal interaction in computing mismatch responses (e.g., prediction errors).

Furthermore, human fMRI studies based on the mental imagery paradigm also investigate the PP mechanism within cortico-cortical interactions associated with declarative memory (Chu et al., 2023; Ortiz-Tudela et al., 2023). These studies support the differentiated mechanism between memory and motor-based predictions (Chu et al., 2023), and episodic and semantic predictions (Ortiz-Tudela et al., 2023).

Together, these studies further show how PP could bridge the field of learning and perception (Aitken & Kok, 2022; K. Friston, 2018), and capture declarative memory function.

### 2.5. Cognitive control

In order to reach our desired goal, we need to adjust our behavior by using our perception, knowledge, and goals to bias the selection of actions and thoughts from multiple choices (Gazzaniga et al., 2019). These processes are called cognitive control or executive function, essential for our intelligent behavior (Miller, 2000). Converging studies showed that the prefrontal cortex (PFC) networks including the frontoparietal (FPN) network, the cingulo-opercular network (CON), the salience network (SN), the default mode network (DMN), and the dorsal and ventral attention networks (DAN and VAN) are central to these processes (Menon & D’Esposito, 2022; Miller, 2000); Yet there is a clear need for a unifying framework for interpreting these varieties of PFC networks supporting cognitive control functions.

Active inference holds a unified view of functional brain architectures and suggests that multiple behavioral controllers (i.e., pavlovian, habitual, and goal-directed) can be understood as the successive contextualizing basic sensorimotor mechanisms within hierarchical generative models (Pezzulo et al., 2015). The achievement of goals and fulfilling drives require suppressing various types of prediction errors (including interoceptive, proprioceptive, and exteroceptive errors) in the hierarchical architecture and resolving them through appropriate actions (Pezzulo et al., 2015). Active Inference considers control as distributed processes across a continuous spectrum ranging from abstract, forward-looking, and conscious reasoning at the highest levels (e.g., PFC) to concrete, nearsighted unconscious reasoning at lower levels, extending to the arc reflex (Pezzulo et al., 2015). In this scenario, cortical nodes in the FPN network, including dorsolateral PFC, induce top-down biases to lower areas, which permits higher-level goals to bias sensorimotor competition and to exert cognitive control (Pezzulo et al., 2018). Meanwhile, cortical nodes in the CON and SN network, including the insula, hypothalamus, the solitary nucleus, and the amygdala, sets the precision of top-down signals (Pezzulo et al., 2018). Finally, the attentional networks play a crucial role in managing the equilibrium between higher-level cognitively intricate goals and more fundamental goals maintained at various hierarchical levels (Pezzulo et al., 2015). This balance represents a significant characteristic of cognitive control.

Another PP-based model from Alexander and Brown suggested a unifying model that incorporates hierarchical predictive coding interaction between FPN and CON networks supporting varieties of cognitive control functions including goal selection, maintenance, and performance monitoring (Alexander & Brown, 2011, 2015, 2018, 2019). As discussed in the Working memory section, the HER model tries to capture the hierarchical function of ACC/mPFC and dlPFC/mPFC with the hierarchical iterative motif of prediction and prediction error computation (Alexander & Brown, 2015, 2018, 2019). The assumption of hierarchical predictive coding between FPN and CON networks, recently supported with an offline TMS-fMRI study that showed cTBS over mid-dlPFC increased both CON and FPN activity down to the hierarchy (Wood et al., 2024).

In sum, the (precision-weighted) prediction error minimization principle can be applied not only to solve lower-level processes in a hierarchy but also to explain cognitive control functions. This principle offers an excellent opportunity to investigate how information from lower levels of the hierarchy contributes to higher-level decision-making processes, such as cognitive control (See (Verguts, 2017)). However, these explanations are still largely hypothetical and need more investigations to compare their explanatory power to other descriptive or phenomenological models in cognitive control, such as reinforcement learning or drift-diffusion models (Sprevak & Smith, 2023).

### 2.6. Language

Human language involves a multistage computational process that transforms thoughts into auditory signals and vice versa (Hickok, 2009).

Decades of experimental work show that processing linguistic stimuli is highly context-dependent, and predictability of upcoming stimulus facilitates language processing while deviating from expectation increases processing time and costs (Altmann & Kamide, 1999; Balota et al., 1985; McDonald & Shillcock, 2003; R. K. Morris, 1994). These contextual predictions come from all stages of linguistic hierarchy including speech sounds, words, and sentences (Ferreira & Chantavarin, 2018; Ferreira & Qiu, 2021; Huettig, 2015; Nieuwland, 2019; Tavano & Scharinger, 2015). In that sense, PP could well explain language comprehension, and language production (speech) in the human brain by considering the hierarchical directional message-passing of predictions between lower-order sensory (i.e., auditory signals), motor (i.e., motor commands) and higher-order cognitive levels (thoughts) (Tavano & Scharinger, 2015).

The coordinated temporal interplay between the inferior frontal gyrus (IFG), superior temporal gyrus and sulcus (STS), and angular gyrus (AG) is suggested to play an important role in language comprehension (Obleser & Kotz, 2010; Schroën et al., 2023). PP mechanism between these core regions in language comprehension is well supported by multiple studies. For instance, MEG studies support the top-down predictive mechanism of left IFG (Liu et al., 2020) and bottom-up prediction error activity in the STG (Gagnepain et al., 2012) during expected versus unexpected speech processing. A recent online TMS-EEG study reveals their precise Causal temporal interaction (Schroën et al., 2023), which underscores the top-down influence of the left IFG on the left STG during the processing of highly predictive verbs within a 150 to 350 ms time window, alongside a bottom-up activity from the left STG to the IFG within a 300 to 500 ms time frame (Schroën et al., 2023). Moreover, another TMS study also supports the contribution of top-down predictive activity from the angular gyrus when bottom-up sensory signals are degraded (Hartwigsen et al., 2015).

Towards a mechanistic understanding of this interplay, Caucheteux et al. (2023) fit deep language algorithms with long-range predictions to fMRI brain activation of 304 participants while listening to spoken language. They demonstrated that prediction in language processing organizes hierarchically in multiple timescales; STG predicts lower-level syntactic representations, while IFG and angular gyrus predict high-level semantic representations. Together these results align with the functionally and temporally distinct pathway of prediction and prediction error in the human language network supporting language comprehension.

In language production, PP involves predicting the sensory consequences of motor commands through motor-to-sensory neural projections, which contribute to detecting and correcting errors in motor control (Okada et al., 2018). Neural evidence supporting the presence of the PP mechanism in speech-motor control has been demonstrated through experiments contrasting internally and externally generated speech. Empirical investigations utilizing MEG (Tremblay et al., 2003) and ECoG (Forseth et al., 2020) reveal that unlike internally generated speech (e.g., reading), externally produced speech (e.g., speaking) suppresses activity in auditory sensory regions, which represent forward predictions. Moreover, overtly articulated speech, as opposed to imagined speech, enhances response in sensory areas as measured by fMRI, reflecting an increase in prediction error (Okada et al., 2018).

Furthermore, PP is not just restricted to spoken language; even in a deaf population using sign language for communication, semantic predictions also exist (Wienholz & Lieberman, 2019). This shows that there are predictive mechanisms for sign language processing in the visual modality and further suggests that PP is a modality-independent property in language processing (Radošević et al., 2022).

### 2.7. Summary so far

The RDoC project is dedicated to exploring mental health disorders through the lens of neuroscience advancements. Current constructs relied on our circuit-based understanding of psychological processes and their relationship to clinical syndromes. Meanwhile, RDoC is an evolving tool and calls for computational models to validate their constructs (Cuthbert, 2022). To understand the differences between domains and their relations based on their underlying neural circuity.

Researchers have criticized the RDoC matrix for lacking a clear rationale or systematic foundation, with no robust path to external validation (Ross & Margolis, 2019). Generally, RDoC suffers from a holistic view of human cognition (Lange et al., 2021). In that sense, we find the explanatory power of the PP framework as the best option, to be a groundwork in understanding RDoC constructs and their overlapping neural circuity. Within this framework, the RDoC matrix could go beyond clustering constructs with correlational studies. This represents a pathway towards a mechanistic understanding of psychological processes, or causality, which consolidates various psychological phenomena under the overarching principle of (precision-weighted) prediction error minimization.

Nevertheless, the explanatory potential of PP requires empirical testing. Although there is compelling evidence supporting PP in the perception construct and its underlying neural circuitry, empirical human studies investigating PP in other constructs within the cognitive system domain are notably scarce.

The first reason for this limited evidence is the lack of methodology in testing PP claims in human studies (Walsh et al., 2020). Although RDoC emphasizes human studies for validating constructs, the current methodology used in human studies could not bring a definite answer to the existence of prediction and prediction error units in different processing hierarchies supporting different psychological processes (Walsh et al., 2020). That’s why using other non-invasive methods like optogenetic, calcium imaging, and single-unit recording in non-human studies is suggested, which directly evaluates the PP hypothesis and makes refinements in neural circuity supporting prediction error minimization (Keller & Mrsic-Flogel, 2018; Walsh et al., 2020). However, we believe emerging techniques like ultra-high-field fMRI and non-invasive brain stimulation techniques like TMS could enhance our understanding of PP in constructs within the cognitive system domain. Ultra-high-field fMRI could distinguish between bottom-up and top-down cognitive processes, offering deeper insights into the laminar circuitry of PP underlying various psychological processes (Haarsma et al., 2022). Moreover, integrating this technique with non-invasive brain stimulation methods, such as TMS, offers a unique opportunity to causally test PP claims on specific neural circuits within constructs. This combination could lead to a more nuanced understanding of how PP mechanisms operate in each brain hierarchy.

Second, while simulation studies based on active inference provide promising formal explanations for different psychological processes related to decision-making and action selection, empirical support for these explanations is limited (see (Hodson et al., 2024)). Indeed, empirical evidence for active inference has been carried out primarily within the context of computational psychiatry for describing mechanisms underlying psychopathologies, rather than describing the brain functions itself (Hodson et al., 2024; Smith et al., 2021).

Together, although PP models present a promising framework for understanding efficient information processing in the human brain, the current evidence does not fully support their utility for validating RDoC constructs. We strongly suggest empirical testing of PP explanations (predictive coding and active inference), and their translation from theoretical algorithms to concrete biophysical implementations within each construct. Only in that case, validating RDoC constructs based on underlying neural circuity with the PP framework is possible.

## 3. Multi-System Integration with Predictive Processing

Psychiatric disorders encompass complex interactions among genes, molecules, cells, circuits, physiology, and behaviors. The RDoC project encourages scientists to acquire data in different units of analysis in each functional domain beyond the categorization boundaries of DSM or ICD (Cuthbert, 2022). Crucially, the integration between these units of analysis is essential for a comprehensive understanding of mental disorders, and the application of computational psychiatry plays a pivotal role in deciphering the complex, dynamic interrelations among these varied dimensions (Sanislow et al., 2019).

In the previous section, we reviewed empirical studies supporting PP in psychological processes within the cognitive system domain. Here, we talk in favor of its power in integrating data from different units of analysis while considering developmental trajectories and environmental factors in psychopathologies.

**Figure 1:**
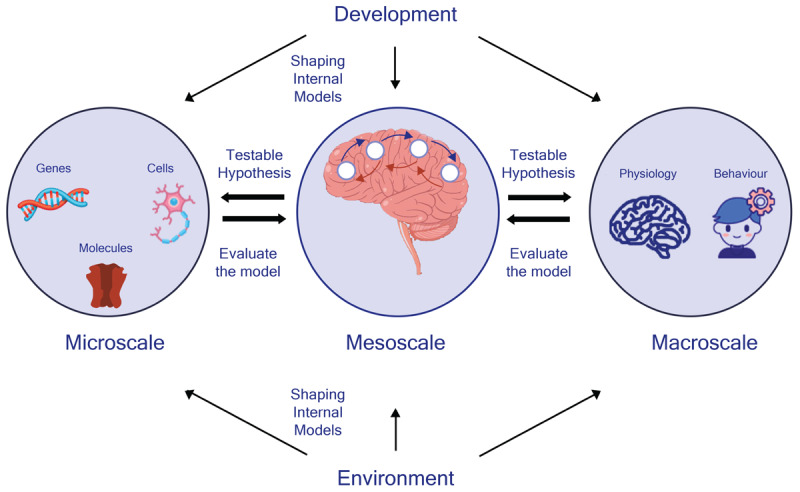
RDoC matric through the lens of Predictive Processing. Computational models based on a predictive processing framework provide mesoscale insight into psychopathologies, generating testable hypotheses regarding data produced in different units. Furthermore, psychopathologies may be conceptualized as the aberrant encoding of internal models, influenced by developmental and environmental factors.

What is unique about the PP framework is its power to formulate hypotheses (Pezzulo et al., 2024). These hypotheses make specific empirical predictions that span different units of analysis, ranging from gene to behavior, which can be empirically validated. One way toward this validation involves formalizing these hypotheses into generative models. By fitting these models to measured data, we can systematically compare them to alternative or competing hypotheses and assess their explanation (Figure 1).

At the physiological level, PP has empirical predictions about the top-down and bottom-up dynamics supporting prediction and prediction error under the name of predictive coding (K. Friston, 2018). Additionally, it elucidates how motor functions (e.g., oculomotor performance) and higher-level cognitive processes (e.g., planning) manifest as an active form of prediction error minimization, under the name of active inference (Parr & Friston, 2018; Pezzulo et al., 2018, 2024).

At the circuit level, the exchange of information between top-down and bottom-up pathways can be understood through oscillatory dynamics within and across brain areas, potentially indicating temporal predictions (Bastos et al., 2012; Walsh et al., 2020). On the molecular and cellular levels, synaptic activity and efficacy, modulated by neuromodulators, correspond to inferential processes that minimize free energy across faster and slower time scales, respectively (K. Friston, 2023; Parr & Friston, 2018; Pezzulo et al., 2024). This involves precision dynamics, which balance inferential processes at multiple levels by adjusting the post-synaptic gain of sensory or prediction error units (K. Friston, 2023). Finally, the PP framework can interpret genetic findings from Genome-Wide Association Studies (GWAS) and Transcriptome-Wide Association Studies (TWASs), particularly when we find its associations with prediction error signals like Mismatch Negativity (MMN) (Bhat et al., 2021; Herzog et al., 2023).

What this broad explanatory power could offer to the RDoC matrix is interpreting data gathered in different units of analysis under the one unifying principle (K. Friston, 2023): minimizing free energy. In other words, for each construct, multiple hypotheses could emerge under the PP framework in different units of analysis, and each of them tested empirically to find the best explanation for the underlying mechanism (Figure 1).

Another critical concern of the RDoC matrix is integrating developmental processes and their interactions with environmental factors (Cuthbert & Insel, 2013; S. E. Morris et al., 2022). In terms of PP, top-down predictive information could emerge while the agency continually faces persistent statistical regularities of the natural environment; otherwise, it could be hardwired in the first place due to phylogenic development (Walsh et al., 2020). Animal studies showed that exposure to statistical regularities of visual stimuli in the course of the experiment results in the emergence of predictive activity (Attinger et al., 2017; Berkes et al., 2011; Fiser et al., 2016; Gavornik & Bear, 2014); aligned with animal studies, neurophysiological studies with human participants also showed a sustained increase in neural activity when auditory stimuli transit from random to regular sequences (Auksztulewicz et al., 2017; Barascud et al., 2016; Southwell & Chait, 2018). Interestingly, recent studies showed that even infants can predict basic contingencies of the environment by employing statistical learning principles (Köster et al., 2020). In this regard, extracting statistical regularities while exploring the environment leads to learning and updating our internal model of the world, and this also includes the infant’s early development and learning (Köster et al., 2020).

Together, the dynamic interaction between developmental trajectories and environmental influences leads to shaping our internal models and priors across multiple levels (Figure 1). Designing experiments that target the influence of these variables on construct-based PP processes could unravel the interaction between these factors and the aberrant encoding of internal models (or precision) associated with psychopathological symptoms.

## 4. Integrating RDoC and PP: A Framework for Understanding Psychotic Disorders

**Figure 2:**
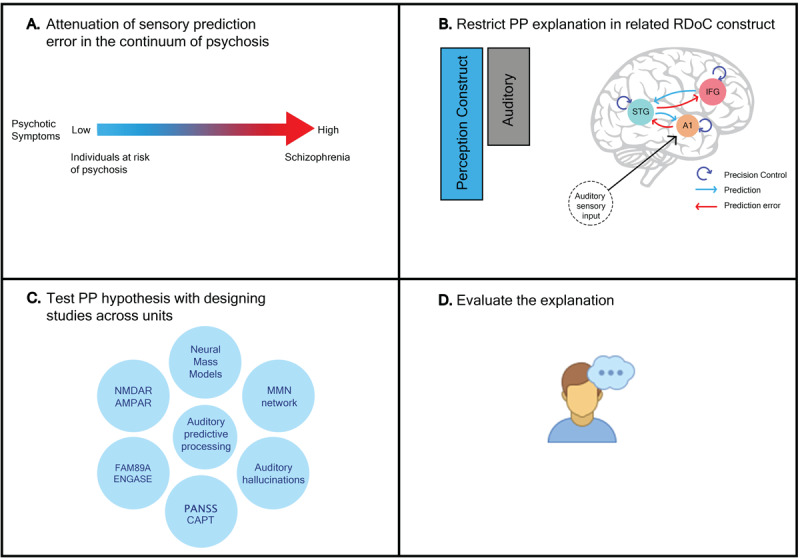
Integrating units of analysis with predictive processing framework across psychosis continuum. A. Attenuation of mismatch negativity (MMN) illustrates aberrant sensory prediction error across the psychosis spectrum, from healthy individuals with psychosis-like experiences to those with established psychotic disorders. B. predictive processing framework generates testable hypotheses for specific RDoC constructs, exemplified here by the auditory MMN circuit within the perception construct. C. Hypothesis are investigated using predictive processing paradigms, leveraging generative models to integrate data across molecular, cellular, and physiological levels. In this context, our example connects auditory predictive processing to a spectrum of biomarkers, each reflecting different units of analysis from genes to behavior. D. Predictive processing explanations are then revised based on empirical data.

Numerous studies have employed the PP framework to explain psychopathological symptoms, yet integrating PP with the RDoC needs a more structured experimental design. This process could begin with identifying an interpretable physiological component that could explained in terms of the PP framework (e.g., MMN as a prediction error) that demonstrates variability across a spectrum of psychiatric conditions (Randeniya et al., 2018) (Figure 2A). This spectrum includes individuals at high risk of a condition as well as those with established psychiatric disorders (Larsen et al., 2020; Randeniya et al., 2018).

The PP framework’s explanatory potential can then be applied to explaining the psychopathological symptoms and generate testable hypotheses about their underlying mechanisms. These hypotheses are formulated as conceptual models restricted to the specific construct of the RDoC (Figure 2B). To test these hypotheses, one could design an experiment to target the network underlying corresponding construct (e.g., classic auditory oddball for perception construct) and then use generative models of brain responses, fit these models to empirical data (such as EEG and fMRI), estimate the model parameters and infer unobservable biological components within a target network (K. J. Friston et al., 2003) (Figure 2C).

DCM is a prominent approach for testing PP-based hypotheses. The field of neural mass models within DCM for electrophysiological signals could offer insights beyond simple feedforward and feedback connections, and model extrinsic and intrinsic connectivities by representing three to four population neuronal models in each region (Pereira et al., 2021). This approach facilitates linking various units of analysis including physiology, circuit, and cellular levels in human studies. Furthermore, genetic findings could be taken into account when we find their associations with prediction error signals like Mismatch Negativity (MMN) (Bhat et al., 2021; Herzog et al., 2023). These hypotheses could be evaluated and refined with respect to empirical data coming from different units of analysis (Figure 2D). While we primarily discuss human studies, advances in neuroscience research of PP in animal studies could translate to PP-based explanations and related computational models (Bastos et al., 2020; Keller & Mrsic-Flogel, 2018; O’Toole et al., 2023).

Several recent studies have embarked on this path, indicating a promising trend for future research in this field. We now turn our focus to these studies, illustrating the utility of the PP framework in explaining psychotic symptoms. Following the RDoC guidelines, these studies offer insights into different units of analysis, providing mechanistic explanations of complex psychiatric phenomena.

Psychotic disorders (e.g., bipolar affective disorder and schizoaffective disorder) and schizophrenia share genetic risk variants, neurobiological abnormalities, cognitive dysfunctions, and patterns of symptoms (Cuthbert & Morris, 2021). Various biomarkers for psychosis exist in different units of analysis, including genetic biomarkers (Allen et al., 2020), neurophysiological biomarkers (Ford et al., 2020; Wang et al., 2022), brain imaging biomarkers (Lahti & Kraguljac, 2020; Lyall et al., 2020; Pearlson & Stevens, 2020), and cognitive biomarkers (Hill et al., 2020). One way toward multi-system integration is by using PP explanatory power to explain the interaction between these biomarkers.

As said, we could start with an interpretable physiological component in terms of the PP framework that varies across a dimension of psychiatric conditions. In the case of psychosis, we could see the mismatch negativity deficit across the spectrum, ranging from individuals at high risk for psychosis to those with established psychotic disorders (Randeniya et al., 2018). This component, which elicits EEG and MEG signals, is a hallmark of sensory prediction errors (Randeniya et al., 2018). This suggests that the impairment in sensory prediction error processing, as evidenced by MMN reductions, is a marker of psychosis vulnerability rather than a symptom unique to schizophrenia (Randeniya et al., 2018).

The next step is targeting a specific construct. There are different kinds of paradigms that produce MMN responses (Kirihara et al., 2020), that could engage networks related to different constructs within a cognitive system domain (Lee et al., 2017). For instance, in the classic and roving auditory oddball paradigm, participants were instructed to either ignore the presentation of auditory stimuli or engage in a distraction task while listening to repetitive sounds, which aligns with the perception construct as they target pre-attentive processing of auditory prediction errors (Larsen et al., 2024). Consequently, PP can provide explanations and generate testable hypotheses, specifically within the perception construct and auditory modality.

PP hypothesizes that auditory hallucinations come from aberrant precision control of priors (Corlett et al., 2019). In a study of a large cohort consisting of 116 schizophrenia and schizoaffective disorder cases, 75 bipolar and major depressive disorder cases, and 248 non-psychotic disorders, Donaldson et al. (2020) found a negative correlation between auditory hallucinations, as measured by the Scale for the Assessment of Positive Symptoms (SAPS), and mismatch negativity (MMN) duration across all case groups compare to never-psychotic individuals. Complementary, a machine learning study based on the BOLD responses to an auditory oddball task showed that prediction error could predict the severity of hallucinations in schizophrenia patients (Taylor et al., 2020). These suggest that, in the context of the psychosis continuum, diminished auditory prediction error may be linked to increased auditory hallucinations from a physiological, behavioral, and self-reported standpoint.

The generation of frequency and duration MMN in oddball paradigms is linked to neural populations in the A1, STG, and IFG (Garrido et al., 2008; Schall et al., 2003). A growing body of evidence now supports the altered effective connectivity between these regions across the continuum of psychosis from a non-clinical population with psychotic-like experiences (Dzafic et al., 2020) to individuals with a genetically high risk of psychosis (Larsen et al., 2018, 2024) to schizophrenia patients at the end of the continuum (Adams et al., 2022; Dzafic et al., 2021; Larsen et al., 2020). More importantly, attenuated connectivity between IFG and STG and intrinsic connectivity in the IFG have been linked to the degree of positive symptoms, including hallucination and delusion (Dzafic et al., 2020; Larsen et al., 2020). The strength of these connections could also depend on the severity of psychopathologies; for instance, Dzafic et al. (2021) demonstrated a decrease in STG to IFG connectivity underpinning auditory prediction errors in individuals with more severe hallucinations.

Another way to explore precision control in PP at the synaptic, cellular, and molecular scales is by using neural mass models (NMM) (Pereira et al., 2021). By parameterizing intrinsic and extrinsic connectivities in the canonical microcircuit (CMC) model and fitting these models to time-series data, we could have a closer look at PP within a target network (K. Friston, 2023). Adams et al. (2022) investigated synaptic efficacy in the MMN network by studying 107 schizophrenia patients, 57 first-degree relatives, and 108 control subjects across various paradigms, including resting state EEG, resting state fMRI, MMN paradigm, and 40-Hz auditory steady-state response (ASSR). They employed parametric empirical bayes in DCM for group-level analysis within and across paradigms that reveal increased self-inhibition in superficial pyramidal cells as a major difference in schizophrenia patients (Adams et al., 2022). While a previous study supports the link between self-inhibition in superficial pyramidal cells and precision weighting (and not prediction error per se) (Lecaignard et al., 2022), this study could further support the aberrant encoding of precision in schizophrenia patients.

Aberrant precision control in psychosis is also linked to the hypofunction of cortical N-methyl-D-aspartate receptors (NMDAR) (Adams et al., 2013). In terms of PP, NMDAR determined synaptic gain and was suggested as a responsible factor for encoding precision (Adams et al., 2014). Thus, altered neurotransmitter function and network dynamics due to NMDAR hypofunction result in the aberrant encoding of precision, leading to increased prediction error and subsequent aberrant learning (Adams et al., 2014; Sterzer et al., 2018). Rosch et al. (2019) explored this idea by analyzing the effects of NMDAR blockade in healthy participants during a roving auditory oddball paradigm under ketamine. This study suggested that ketamine-induced MMN amplitude reduction is linked to intrinsic regional connections, specifically disinhibition in the IFG due to altered interneuron activity. Another computational modeling study further showed the association between the ketamine effect and decreasing higher-level prediction errors in healthy human adults (Weber et al., 2020). These studies offer insights into bridging neurophysiological and biological components, enhancing understanding of ketamine-induced psychotic-like symptoms (Rosch et al., 2019).

Psychotic symptoms also share genetic liabilities across various diagnostic categories (Calabrò et al., 2020; Herzog et al., 2023). Bhat et al. (2021) examined the relationship between gene expression in cortical tissues and MMN peak amplitude in 728 individuals. They found that gene expressions related to MMN, particularly the FAM89A gene in the frontal region and the ENGASE gene in the entire brain, negatively correlated with MMN amplitude (Bhat et al., 2021). These genes mainly decode proteins related to regulating the concentration of neurotransmitters in synaptic clefts (Bhat et al., 2021). Such investigations into gene expressions associated with auditory prediction error hold the potential to develop precise genetic models delineating abnormal modulation of precision, specifically within the perception domain of the RDoC framework, thereby advancing our understanding of psychosis.

Together, we start with an idea of aberrant precision control in the continuum of psychosis. Then we provide a few valuable studies that try to understand this aberrant control from gene to behavior (see Table 1). In psychosis, a negative correlation was observed between auditory hallucinations and MMN across the entire continuum (Donaldson et al., 2020). DCM studies have revealed associations between forward and backward connections between IFG and STG, increased disinhibition (evidenced by enhanced self-inhibition in the superficial pyramidal cells and subsequent downregulation of interneurons) in IFG, and the presence of positive symptoms (Adams et al., 2022; Larsen et al., 2020, 2024; Rosch et al., 2019). Furthermore, a transcriptomic study has uncovered a negative correlation between the expression of two genes in the adult human cortex and MMN amplitude, indicating a need for further research to elucidate its connection to the synaptic and cellular dysfunctions observed in psychosis (Bhat et al., 2021).

**Table 1:** Recent Studies Examining Mismatch Negativity (MMN) as an Index of Auditory Prediction Error Across Various Units of Analysis.


	GENES	MOLECULES	CELLS	CIRCUITS	PHYSIOLOGY	BEHAVIOR	SELF-REPORTS	PARADIGM

Larsen et al. (2024)			NMM	MMN Network	EEG		CAPE, PANSS	Auditory oddball

Larsen et al. (2020)			NMM	MMN Network	EEG		CAPE, PANSS	Stochastic mismatch negativity

Dzafic et al. (2021)			NMM	MMN Network	EEG	Statistical learning	CAPE, PANSS	Reversal auditory oddball

Dzafic et al. (2020)			NMM	MMN Network	EEG	Statistical learning	PQ	Reversal auditory oddball

Larsen et al. (2018)			NMM	MMN Network	EEG		SIPS	Auditory roving oddball

Rosch et al. (2019)		Ketamine	CMC	MMN microcircuit	EEG			Auditory roving oddball

Adams et al. (2022)			CMC	MMN microcircuit	EEG, fMRI		APSS	Auditory oddball

Bhat et al. (2021)	FAM89A and ENGASE				EEG			Auditory oddball

Donaldson et al. (2020)					EEG		SAPS	Auditory oddball

Taylor et al. (2020)					fMRI		SAPS, SANS	Auditory oddball

Weber et al. (2020)		Ketamine			EEG	Statistical learning		Auditory roving oddball


*Note*. Abbreviations: CAPE = Community Assessment of Psychic Experiences; MMN = Mismatch Negativity; NMM = Neural Mass Models; CMC = Canonical Microcircuit; PANSS = Positive and Negative Syndrome Scale; SAPS = Scale for Assessment of Positive Symptoms; SANS = Scale for Assessment of Negative Symptoms; APSS = Auditory Perceptual State Score; SIPS = Structured Interview for Prodromal Symptoms, PQ = Prodromal Questionnaire.

We believe the claims of RDoC about the potentials of data-driven models (e.g., B-SNIP project), in providing neurobiological targets for treatment development (Clementz et al., 2016, 2022; S. E. Morris et al., 2020) are not sufficient as these models could not offer a mechanistic understanding of molecular and regional connections between biomarkers and clinical characteristics (K. Friston, 2023). The PP framework, at least in some constructs with known underlying neural circuity, could link these chains of evidence together to find a mechanistic implication for psychotic symptoms, which could finally lead to reliable targets for treatment development.

## 5. Conclusion

The Research Domain Criteria (RDoC) framework significantly relies on computational models for two primary objectives: first, to evaluate and validate its constructs by focusing on their underlying neural circuitry, and second, to integrate different units of analysis by considering developmental trajectories and environmental influences associated with psychopathologies. However, the RDoC funding project mostly relies on data-driven approaches and ignores the most influential theory in cognitive neuroscience.

A multitude of human and non-human studies over the past two decades, employing diverse spatiotemporal scales such as neuroimaging, EEG, and extra-cellular recording, consistently demonstrate that the human brain leverages prediction and subsequent prediction error for efficient information processing. Still, from 120 projects funded by RDoC since its beginning, we only found one project directly focusing on the PP framework (NIMH 2024). This project reveals new insights about the predictive coding accounts for psychosis symptoms in broad clinical and non-clinical groups, including healthy individuals with psychotic-like experience (Corlett et al., 2023), clinical and non-clinical voice hearers (Gold et al., 2023; Leptourgos et al., 2022), and trauma-related hallucinations (Lyndon & Corlett, 2020).

In this review, we tried to assess the potential of PP in addressing the two primary purposes. In the first section of our study, we focus on the first objective of the RDoC, which involves validating constructs. We aim to accomplish this by demonstrating how PP enhances our understanding of constructs within the Cognitive Systems domain at the mesoscale level. To support our assertions, we present empirical evidence from human studies that corroborates the explanatory power of PP.

While PP shows significant explanatory potential within the Cognitive System Domain, empirical evidence in humans is largely lacking, with some exceptions of certain constructs (e.g., Perception and Language). Based on this limited evidence, PP could not be effective for validating RDoC constructs based on their underlying neural circuity. However, we believe future studies utilizing multi-modal data acquisition methods, such as TMS-fMRI, could test the PP hypothesis in human studies based on neural circuities underlying each construct and be valuable in validating the RDoC constructs.

In the second section, we evaluate PP’s potential for integrating different units of analysis. In cases where PP encompasses both interpretable physiological components (e.g., MMN as a prediction error) and known underlying circuity (e.g., MMN network), it demonstrates a strong capability for connecting different units of analysis. We highlight a few valuable studies that follow this approach, contributing to our understanding of the mechanistic interactions among these units in psychosis.

It is worth noting that PP studies that try to explain psychopathologies heavily rely on the DSM categorization system (see (K. J. Friston, 2017)). Systematic reviews that evaluate PP studies in mental health disorders mostly come with mixed results (Angeletos Chrysaitis & Seriès, 2023; Cannon et al., 2021). In one of these systematic reviews, the authors declared, “These ambiguities cannot be resolved without a clear framework for the hierarchy of priors in the brain and possibly its implementation in computational models” (Angeletos Chrysaitis & Seriès, 2023). In that sense, we believe PP itself needs the RDoC framework to study psychopathologies multi-dimensionally based on the circuit-based understanding of mental constructs.

In sum, these two lines of research heavily need each other to fill their gaps, toward reaching the ultimate goal of psychiatry, “precision psychiatry” that both pursue.
